# Severe pneumonia caused by *Parvimonas micra*: a case report

**DOI:** 10.1186/s12879-021-06058-y

**Published:** 2021-04-17

**Authors:** Qing Yu, Lingling Sun, Zuqing Xu, Lumei Fan, Yunbo Du

**Affiliations:** 1 Department of Intense Care Unit, Shenzhen Longhua Central Hospital, Guanlan Boulevard, Shenzhen, 187 China; 2 Department of Anesthesiology, Huazhong University of Science and Technology Union Shenzhen Hospital, Taoyuan Road 89, Shenzhen, China

**Keywords:** *Parvimonas micra*, Severe pneumonia, Next-generation sequencing, Case report

## Abstract

**Background:**

*Parvimonas micra (P. micra)* is a gram-positive anaerobic coccus that is detected widely on the skin, in the oral mucosa and in the gastrointestinal tract. In certain circumstances, *P. micra* can cause abdominal abscesses, bacteraemia and other infections. To the best of our knowledge, there have been no case reports describing the biological characteristics of *P. micra*-related pneumonia. These bacteria do not always multiply in an aerobic organ, such as the lung, and they could be easily overlooked because of the clinical mindset.

**Case presentation:**

A 35-year-old pregnant woman was admitted to the emergency department 4 weeks prior to her due date who was exhibiting 5 points on the Glasgow coma scale. A computed tomography (CT) scan showed a massive haemorrhage in her left basal ganglia. She underwent a caesarean section and brain surgery before being admitted to the ICU. She soon developed severe pneumonia and hypoxemia. Given that multiple sputum cultures were negative, the patient’s bronchoalveolar lavage fluid was submitted for next-generation sequencing (NGS) to determine the pathogen responsible for the pneumonia; as a result, *P. micra* was determined to be the causative pathogen. Accordingly the antibiotic therapy was altered and the pneumonia improved.

**Conclusion:**

In this case, we demonstrated severe pneumonia caused by the anaerobic organism *P. micra*, and the patient benefited from receiving the correct antibiotic. NGS was used as a method of quick diagnosis when sputum culture failed to distinguish the pathogen.

## Background

*Parvimonas micra* (*P. micra*), also known as *Peptostreptococcus micros* and *Micromonas micra,* can be commonly detected on the surface of human skin and as a part of the dental and gastrointestinal flora. We have found multiple case reports on *P. micra*-related periodontitis, pylephlebitis [1], iliopsoas abscess [2], abdominal abscess [3], and arthritis [4]. However, we have not found any reports on *P. micra*-related pneumonia. Because the lung is an oxygen-containing organ, it is an unsuitable environment for fastidious *Parvimonas micra*.

Due to difficulty in culturing the bacteria, *P. micra* infection can be missed and thus its treatment delayed, possibly resulting in a worse prognosis [4]. In this case, we used next-generation sequencing (NGS) to detect *P. micra*-related pneumonia.

## Case presentation

A 35-year-old pregnant woman was admitted to our emergency room 4 weeks prior to her due date because of a sudden headache and unconsciousness. Computed tomography (CT) showed a massive cerebral haemorrhage in the right hemisphere, and she was immediately taken to the operation room for an emergency caesarean section. After the foetus was delivered, she underwent a craniotomy to remove the cerebral haematoma. After 8 h of surgery, the patient was taken to the ICU for further treatment.

**Fig. 1:**
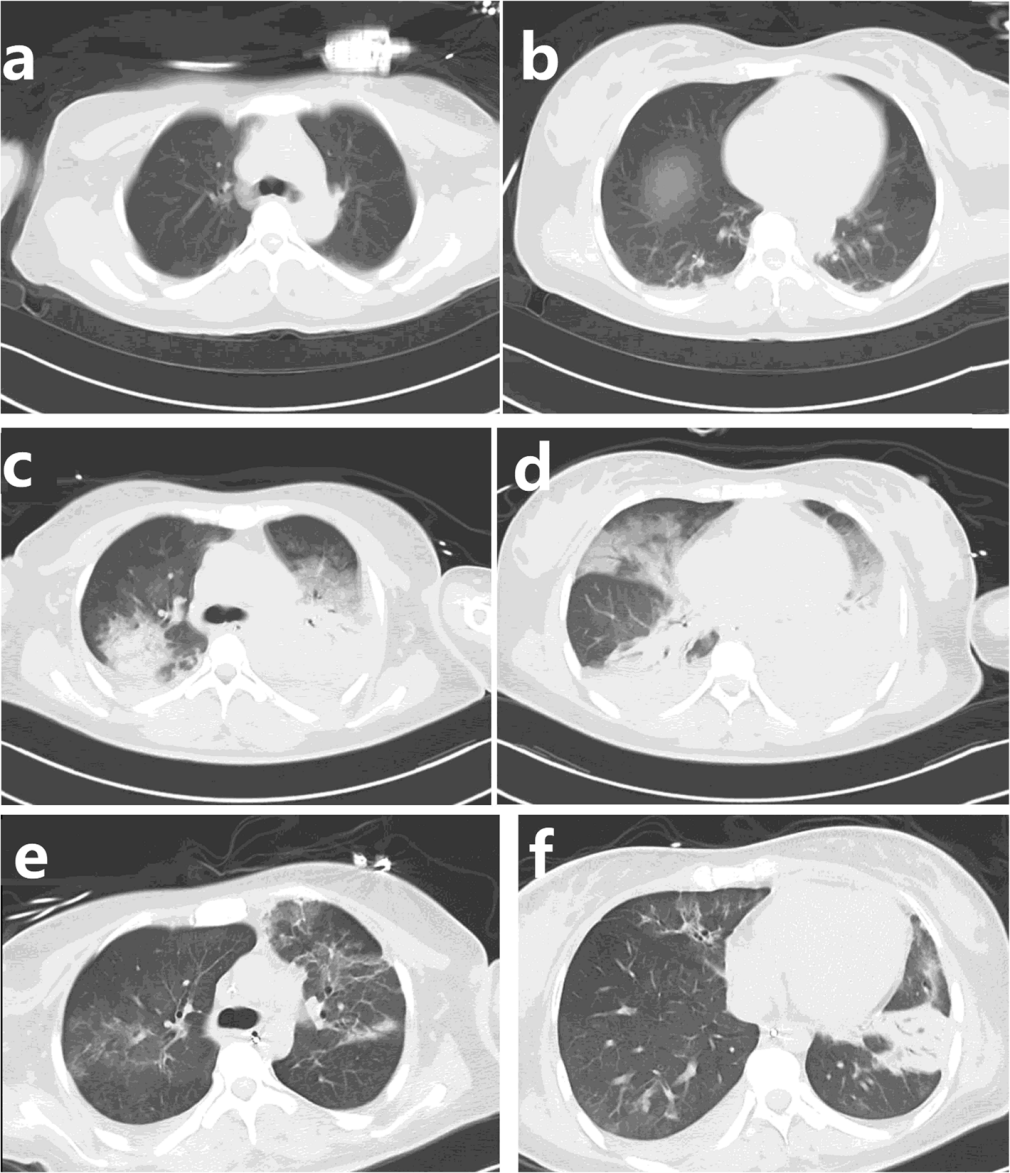
CT findings of lung: **a** and **b** The computed tomography (CT) showed mild exudation in the lower lobe of the lung which revealed a pneumonia at admission. **c** and **d** The CT on day 12 showed the involvement of the lung had progressed. **e** and **f** The CT scan on day 24 showed clearer lung field and less infiltration and atelectasis, and the pneumonia was improved

The patient developed dilated pupils without a pupillary response. The CT scan showed mild pneumonia (Fig. 1a and b) on day 1. Moxifloxacin was started to treat her pneumonia and to prevent surgical site infections. Laboratory examinations on admission revealed a leukocyte count of 10*10^9/ml and a C-reactive protein level of 21.9 mg/L. As the pneumonia progressed, the patient developed febrile fever with a temperature between 38.5–39.5 °C and developed more grossly purulent tracheobronchial secretions, and laboratory studies showed an increased leukocyte count. Meanwhile, no pathogen was found as multiple sputum cultures, blood cultures and IgM for *Chlamydia* and *Mycoplasma* all came back negative, which ruled out common hospital acquired pneumonia caused by *Streptococcus pneumoniae, Klebsiella pneumoniae* or *Staphylococcus aureus*. Despite starting moxifloxacin at admission and meropenem and vancomycin on day 3 after admission, her pneumonia worsened, and she soon developed hypoxemia (oxygenation index 55–85 mmHg). The CT performed on day 12 showed more exudation and atelectasis than previously (Fig. 1c and d).

**Fig. 2:**
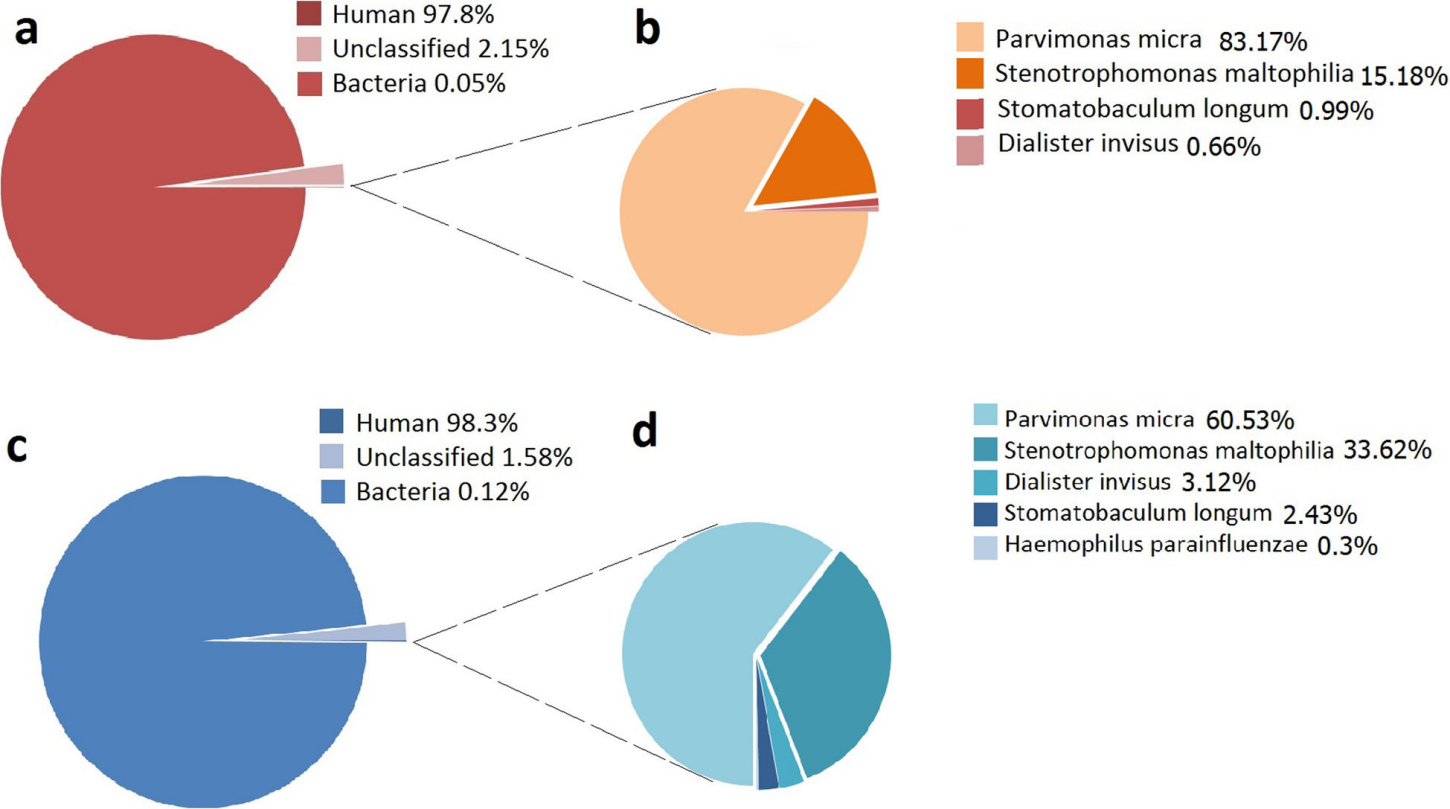
NGS findings on day 13: **a** NGS detected the total DNA reads, 97.8% of the reads were from the human host, 2.15% of the reads were too small to be classified, only 0.05% of the reads were bacterial. **b** Among the bacterial DNA reads, 83.17% of the reads indicated *P. micra* infection. **c** NGS detected the total RNA reads, 98.3% of the reads were from the human host, 1.58% of the reads were unclassified, 0.05% reads were bacterial. **d** 60.53% of the bacterial RNA reads indicated *P. micra* infection

Since the bacteriological culturing protocol failed to demonstrate a responsible pathogen and our current antibiotic therapy failed to control the pneumonia, we came to realize that the pathogen could be an uncommon pathogen for severe pneumonia. We sent a sample of bronchoalveolar lavage fluid for pathogen detection by next-generation sequencing (NGS) at BGI-Shenzhen on day 13 after admission. The NGS test was performed on the BGISEQ-500 platform [5]. Within 24 h, the NGS results identified 252 DNA sequence reads (out of 18,188,496) and 621 RNA sequence reads (out of 38,214,718) corresponding to *P. micra*. After we eliminated all the sequence reads of the human host and the unclassified reads, *P. micra* reads accounted for 83.17% (DNA) and 60.53% (RNA) of the total microbial reads (Fig. 2). The second pathogen identified by NGS was *Stenotrophomonas maltophilia,* which could be a co-pathogen. Thus, the NGS results suggested that *P. micra* was the main pathogen causing pneumonia. On day 14, we adjusted the antibiotic therapy from meropenem and vancomycin to ornidazole for *P. micra* and sulfamethoxazole for *Stenotrophomonas maltophilia* according to our local antibiotic sensitivity pattern.

After starting ornidazole, a prone position was given and daily bronchoscopic sputum suction, the hypoxemia improved. A CT scan performed on day 24 found less infiltration (Fig. 1e and f) and fewer lesions of atelectasis. Bronchoscopy showed less sputum and less bronchial oedema.

Unfortunately, due to the patient’s massive cerebral haemorrhage, she was diagnosed with brain death based on electroencephalogram (EEG) and her clinical status.

## Discussion and conclusions

*P. micra* is a part of the normal flora of the oral cavity, gastrointestinal tract, genitourinary tract and skin. *P. micra* is not a dominant bacterium in an aerobic environment, and there have been multiple case reports on *P. micra*-related abdominal infection [1–3, 6, 7], spondylodiscitis [8, 9], intracranial infection [2, 10, 11] and bloodstream infection [12–15], but we did not find any case reports describing severe pneumonia caused by *P. micra.* On the other hand, we noticed that *P. micra* could cause deep infection in the elderly patients or patients who have undergone surgery and cancer therapy exist [11, 12, 14, 16], indicating that immune insufficiency and postoperative stress could be risk factors for this infection. Our patient had no history of immune insufficiency or cancer, so we suspected that stress after surgery was the main risk factor for this atypical pneumonia.

In this case, the patient was already in a coma before admission to our ER; hence, aspiration pneumonia caused by bacteria in the oral cavity and gastrointestinal tract was taken into consideration. We placed the patient on a ventilator because she had no spontaneous breathing or cough reflex; her lung was filled with thick sputum that enlarged the volume of respiratory dead space, creating a partial anaerobic environment for *P. micra*. After we identified the pathogen with NGS, we used ornidazole to eliminate *P. micra* and prone position ventilation along with bronchoscopic sputum suction to reduce the volume of respiratory dead space. Once *P. micra* was eliminated and the partial anaerobic environment was improved, the infection was quickly placed under control.

Another bacterium identified by NGS was *Stenotrophomonas maltophilia*, which is a common cause of ICU-acquired pneumonia, especially among immunocompetent patients. After a series of consultations with the aetiologist, we thought *Stenotrophomonas maltophilia* might not be the main cause of this pneumonia because the pneumonia still progressed despite the application of a strong antibacterial therapy including moxifloxacin, meropenem and vancomycin. However, *Stenotrophomonas maltophilia* could still contribute to a co-pathogen according to the NGS results.

There were several limitations in this case report. We did not find any bacteria or fungi in blood or sputum culture, and we also did not find *P. micra,* this could be due to the lack of anaerobic bacteria culture facility in our centre. Second, we did not submit bronchoalveolar lavage fluid for next-generation sequencing (NGS) after ornidazole therapy to confirm whether the *P. micra* was eradicated due to the expensive cost of this test. The diagnosis of *P. micra*-related severe pneumonia was determined by the NGS results, the lack of evidence of other common pathogens, and the curative effect of ornidazole therapy.

In conclusion, identifying the pathogen is the key factor for treating pneumonia. When bacteriological culturing fails, NGS may play an important role in quick and precise diagnosis.

## Data Availability

All data generated or analysed during this study are included in this published article.

## Abbreviations

*P. micra*: Parvimonas micra
CT: Computed tomography
NGS: Next-generation sequencing
EEG: Electroencephalogram

## References

[1] Shinha T, Caine V (2015). Pylephlebitis due to Parvimonas micra [J]. Infect Disease Clin Pract.

[2] Sawai T, Koga S, Ide S (2019). An iliopsoas abscess caused by Parvimonas micra: a case report [J]. J Med Case Rep.

[3] Ang MY, Dymock D, Tan JL (2013). Genome sequence of Parvimonas micra strain A293, isolated from an abdominal abscess from a patient in the United Kingdom [J]. Genome Announc.

[4] Baghban A, Gupta S (2016). Parvimonas micra: a rare cause of native joint septic arthritis.[J]. Anaerobe.

[5] Fang C, Zhong H, Lin Y, Chen B, Han M, Ren H, Lu H, Luber JM, Xia M, Li W, Stein S, Xu X, Zhang W, Drmanac R, Wang J, Yang H, Hammarström L, Kostic AD, Kristiansen K, Li J. Assessment of the cPAS-based BGISEQ-500 platform for metagenomic sequencing. GigaScience. 2018;7(3):1–8. 10.1093/gigascience/gix133. 10.1093/gigascience/gix133 PMC5848809 29293960

[6] Shimada K, Inamatsu T, Yamashiro M (1977). Anaerobic bacteria in biliarydisease in elderly patients. J Infect Dis.

[7] Kim EY, Baek YH, Jung DS (2019). Concomitant Liver and Brain Abscesses Caused by Parvimonas Micra [J]. Korean J Gastroenterol.

[8] Uemura H, Hayakawa K, Shimada K, Tojo M, Nagamatsu M, Miyoshi-Akiyama T, Tamura S, Mesaki K, Yamamoto K, Yanagawa Y, Sugihara J, Kutsuna S, Takeshita N, Shoda N, Hagiwara A, Kirikae T, Ohmagari N. Parvimonas micra as a causative organism of spondylodiscitis: a report of two cases and a literature review. Int J Infect Dis. 2014;23:53–5. 10.1016/j.ijid.2014.02.007. Epub 2014 Mar 26. 10.1016/j.ijid.2014.02.007 24680818

[9] Jones SL, Riordan JW, Glasgow AL, Botes J, Boutlis CS (2015). Two cases of spondylodiscitis caused by Parvimonas micra [J]. Intern Med J.

[10] Prieto R, Callejas-Díaz A, Hassan R, de Vargas AP, López-Pájaro LF. Parvimonas micra: A potential causative pathogen to consider when diagnosing odontogenic brain abscesses. Surg Neurol Int. 2020;11:140. 10.25259/SNI_20_2020. 10.25259/SNI_20_2020 PMC7294173 32547827

[11] Ko JH, Baek JY, Kang CI, Lee WJ, Lee JY, Cho SY, et al. Bacteremic meningitis caused by Parvimonas micra in an immunocompetent host [J]. Anaerobe. 2015;34:161–3. 10.1016/j.anaerobe.2015.05.004. 10.1016/j.anaerobe.2015.05.004 25977161

[12] Carretero RG, LunaHeredia E, OlidVelilla M (2016). Bacteraemia due to Parvimonas micra, a commensal pathogen, in a patient with an oesophageal tumour [J]. Bmj Case Rep.

[13] Tonnara G, Colloca GF (2017). Parvimonas Micra Bloodstream infection in a Patient with Oral Mucositis. Res Rep Med Sci.

[14] Cobo F, Borrego J, Rojo MD (2018). Polymicrobial anaerobic bacteremia due to Atopobium rimae and Parvimonas micra in a patient with cancer [J]. Anaerobe.

[15] García-Hita M, Sigona-Giangreco IA, Rincón-Almanza A (2020). Case report: Parvimonas micra infective endocarditis [J]. Enferm Infecc Microbiol Clín.

[16] Gorospe L , Isabel Bermúdez-coronel-Prats, Carol F. Gómez-Barbosa, et al. Parvimonas micra chest wall abscess following transthoracic lung needle biopsy [J]. Korean J Intern Med, 2014, 29(6):834–837, doi: 10.3904/kjim.2014.29.6.834. 10.3904/kjim.2014.29.6.834 PMC4219977 25378986

